# Resolving Sulfation Posttranslational Modifications on a Peptide Hormone using Nanopores

**DOI:** 10.1021/acsnano.4c09872

**Published:** 2024-10-10

**Authors:** Xiuqi Chen, Jasper W. van de Sande, Justas Ritmejeris, Chenyu Wen, Henry Brinkerhoff, Andrew H. Laszlo, Bauke Albada, Cees Dekker

**Affiliations:** † Department of Bionanoscience, Kavli Institute of Nanoscience Delft, Delft University of Technology, Delft 2629 HZ, The Netherlands; ‡ Laboratory of Organic Chemistry, Wageningen University & Research, Wageningen 6807 WE, The Netherlands; § Department of Physics, University of Washington, Seattle, Washington 98195, United States

**Keywords:** nanopore, peptide fingerprinting, post-translational modifications, single-molecule technique, plant peptide hormone

## Abstract

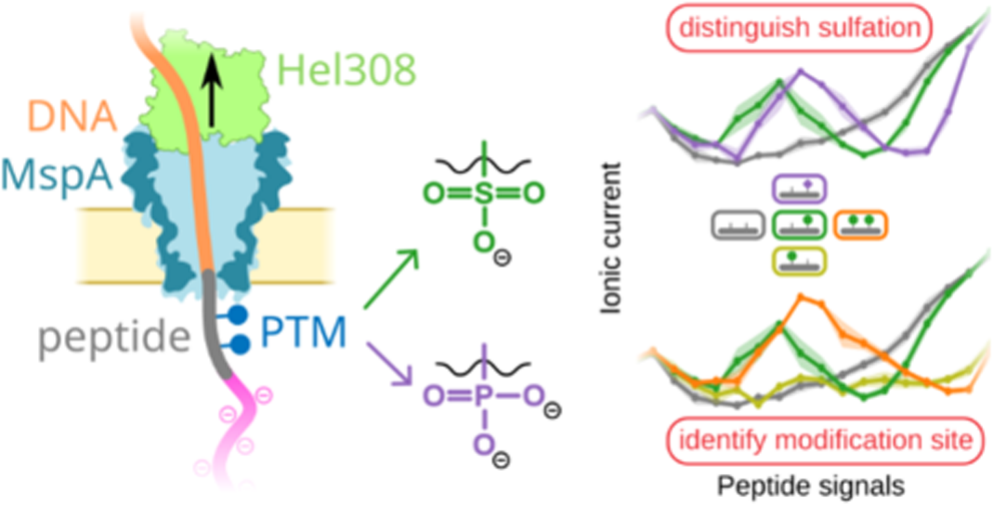

Peptide hormones are decorated with post-translational modifications (PTMs) that are crucial for receptor recognition. Tyrosine sulfation on plant peptide hormones is, for example, essential for plant growth and development. Measuring the occurrence and position of sulfotyrosine is, however, compromised by major technical challenges during isolation and detection. Nanopores can sensitively detect protein PTMs at the single-molecule level. By translocating PTM variants of the plant pentapeptide hormone phytosulfokine (PSK) through a nanopore, we here demonstrate the accurate identification of sulfation and phosphorylation on the two tyrosine residues of PSK. Sulfation can be clearly detected and distinguished (>90%) from phosphorylation on the same residue. Moreover, the presence or absence of PTMs on the two close-by tyrosine residues can be accurately determined (>96% accuracy). Our findings demonstrate the extraordinary sensitivity of nanopore protein measurements, providing a powerful tool for identifying position-specific sulfation on peptide hormones and promising wider applications to identify protein PTMs.

## Introduction

Peptide hormones are essential signaling molecules that mediate intercellular communication.^1^ Many secreted peptides contain at least one post-translational modification (PTM).^2,3^ For example, sulfation and phosphorylation on the tyrosine residue play critical roles in plant signaling pathways and metabolism.^4,5^ For plant peptide hormones such as phytosulfokine (PSK), the presence of a sulfate group on tyrosine is essential for their biological activity.^6^ Specifically, the disulfated PSK pentapeptide (YIYTQ) is active at nanomolar concentrations only when both sulfate groups are present.^7^ Recent bioinformatic estimates indicate that many more tyrosine residues can potentially be sulfated than currently known.^8^ This suggests that sulfotyrosine residues tend to escape observation, likely due to the lability of the sulfoester bond during conventional mass spectrometry workflows, combined with biochemical purification protocols^9^ (Figure S1). Preserving and enriching sulfation PTM during sample preparation is far from optimized with a known bias toward phosphotyrosine,^9−11^ resulting in the underrepresentation of sulfotyrosine occurrence in the peptide phytohormone family. Importantly, the virtually identical masses of phosphotyrosine (79.966 Da modification) and sulfotyrosine (79.957 Da modification), differing by only 0.01 Da, make them difficult to distinguish by mass spectrometry. Furthermore, the exact localization of the sulfation PTM is extremely challenging when multiple potential sites are encountered in a short fragment. This all calls for novel technologies that provide high discriminatory power to differentiate between very similar PTMs and to locate the modification site.

Recent developments in nanopore sequencing technology have provided effective tools for identifying protein PTMs.^12−14^ In this approach, a nanopore is inserted into the lipid bilayer, and a target protein is linearized and slowly translocated through it. Amino acid residues within the pore constriction temporarily block the ionic current in slightly different ways during motor-assisted translocation, and variations in the signals correspond to the composition and sequence of the target protein.^15−17^ Phosphorylation on the protein is known to result in significant ionic current difference in the nanopores,^18−20^ and uncharged PTMs, such as methylation and acetylation, have also been detected.^21^ Because of the localized sensing region, PTMs on different loci often demonstrate distinguishable signals, allowing for accurate positioning.^15,19,20,22,23^ Compared to existing PTM detection methods, such as antibody-based assays or mass spectrometry, nanopore methods are not hindered by antibody bias^24^ or molecular damage during preparation,^9^ while achieving very sensitive detection and localization of PTMs at the single-molecule level.

Here, we reveal how this powerful nanopore sequencing technology can be used to distinguish between sulfotyrosine and very similar phosphotyrosine within the PSK pentapeptide hormone. We find that single PTMs on PSK generate very distinct signals from the unmodified peptide and that sulfation can be accurately distinguished from phosphorylation. The exact location of the modified residue on the two potential PTM sites can also be clearly identified in all permutations at the single-molecule level. We thus show that nanopore sequencing offers a reliable, robust, and accessible method for determining PTMs on peptide hormones with single-molecule sensitivity. Our results provide insights into how charged residues modulate nanopore signals of peptide measurements, marking another essential step toward *de novo* nanopore protein sequencing.

## Results and Discussion

### Translocation of PTM Variants of PSK Peptides through MspA

**Figure 1:**
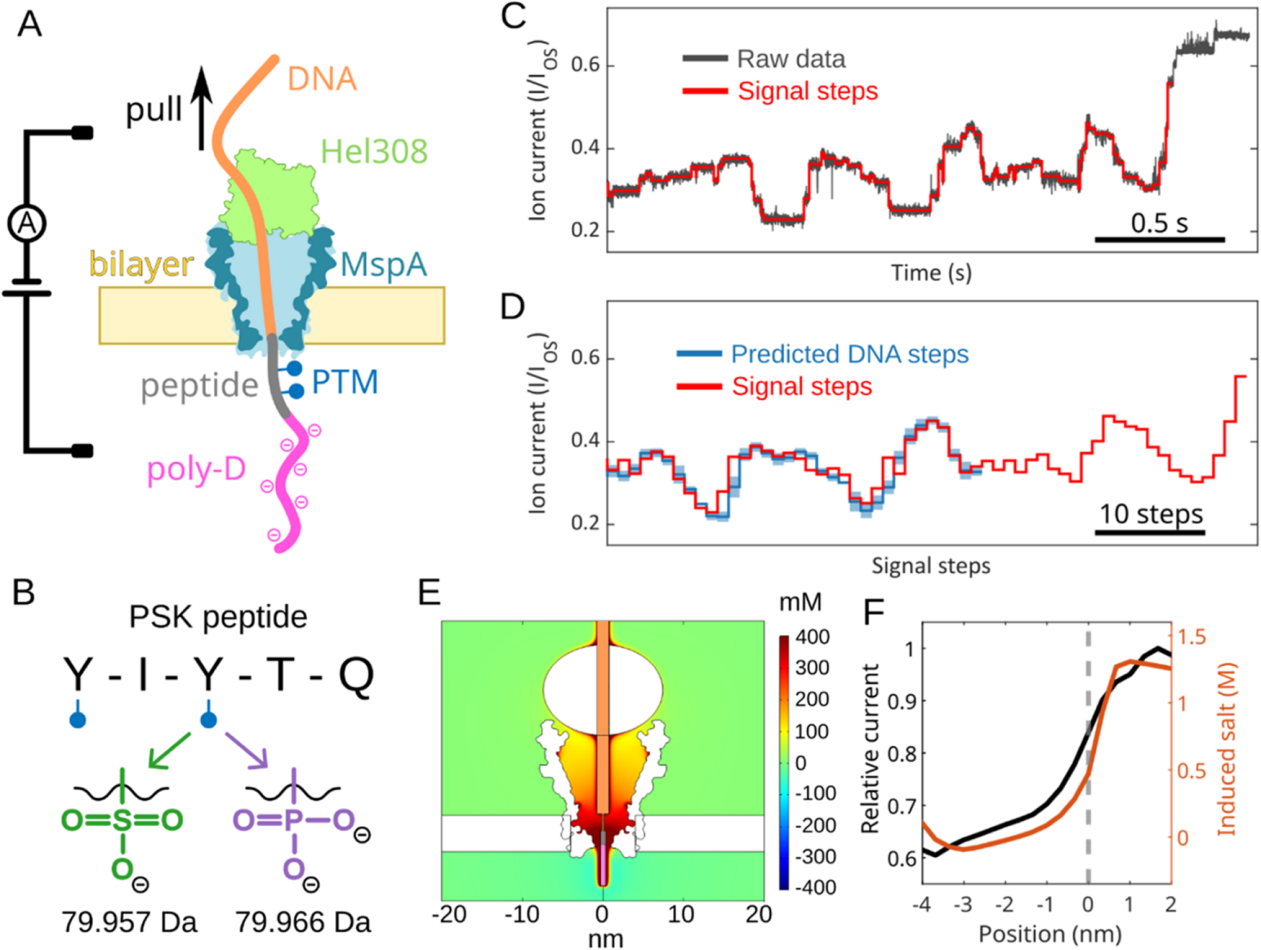
Detecting post-translational modifications on a PSK peptide with nanopore sequencing. (A) DNA-peptide conjugate molecule translocates an MspA nanopore under a voltage bias until the Hel308, which is bound to the DNA, is stuck at the top of the pore. The Hel308 motor protein slowly pulls the DNA upward, generating a stepwise ionic current as the DNA-peptide conjugate passes through the narrow pore constriction. (B) Two tyrosine (Y) residues on the pentapeptide PSK can be modified by either sulfation or phosphorylation, carrying one or two negative charges, respectively. The masses are calculated in their protonated forms. (C) Example ionic current trace from the double sulfation sample. The open-state current (*I*_OS_) of the nanopore is used to normalize the current blockades across different translocation events. Step-like signals are identified and used to characterize the analyte. (D) Given the known DNA sequence, the predicted DNA signals are used to align and segment the full-length signals from the DNA-peptide conjugate molecule. The enzyme occasionally skips and back-steps, creating alignment shifts. (E) COMSOL simulation demonstrates the elevation of local salt concentrations (sum of K^+^ and Cl^–^) near the pore mouth, induced by the densely charged poly-D tail (the central rod models, from top to bottom, the DNA, linker, peptide, and poly-D tail). The color bar on the right denotes the scale of additional salt compared to the normal salt concentration of 400 mM. (F) Correlation of induced additional salt concentration at the nanopore constriction and ionic current during translocation. The dashed line corresponds to panel (E) where the first residue from poly-D tail is positioned at the pore constriction.

To enable the slow and stepwise translocation of the peptide through the MspA (*Mycobacterium smegmatis* porin A) nanopore, one terminus of the peptide is covalently attached to a piece of single-stranded DNA (ssDNA) that is translocated through the pore by a Hel308 helicase (Figure 1A). On the other terminus, we added a negatively charged polyaspartate (D_15_) tail to stretch the molecule under the applied voltage and to improve the efficiency of inserting the peptide into the pore. We synthesized the nine possible sulfation/phosphorylation PSK variants, i.e., no PTM, single PTM on either tyrosine (sulfation or phosphorylation), and PTMs on both residues. These were synthesized on a 15-aspartate chain by a solid-phase peptide synthesis protocol described previously.^25^ The resulting peptides carry an azide group at the N-terminus to enable strain-promoted alkyne–azide click attachment to a piece of ssDNA functionalized with BCN (bicyclononyne; see Methods Section and Figure S2 for the molecular structure). By applying a constant voltage bias across the lipid bilayer, the ionic current through the MspA nanopore was recorded and the translocation of the conjugate molecule was identified by the current blockade. The stepwise translocation induced by the Hel308 motor protein produced signal steps in the ionic current, as described previously^26,27^ (Figure 1C). With the established signal prediction for DNA sequencing,^28^ the onset of the peptide signals could be identified as following the aligned DNA signal steps (Figure 1D).

For all peptide variants in this study, we observed a significant ionic current increase near the end of the translocation event, which served as a consistent reference for thresholding the end of the peptide signals (Figure S3). Finite-element analysis with COMSOL Multiphysics^Ⓡ^ (Figure 1E and Methods Section) showed that this signal ramp can be attributed to the ion enrichment in the nanopore due to the dense negative charges on the poly-D tail. Known as ionic concentration polarization,^29^ the charges on the peptide raise the local ion concentration and hence increase the ionic current when this part of the peptide is located near the nanopore constriction (Figure 1F). The onset of this current increase occurs slightly before the tail starts translocating the nanopore (Figure 1F, dashed line). A recent molecular dynamics (MD) study discussed a similar effect from charged polymers during translocation.^30^ A less densely charged poly-T ssDNA tail resulted in only a minor ionic current elevation (Figure S4).

### Sulfation and Phosphorylation Can be Clearly Distinguished

**Figure 2:**
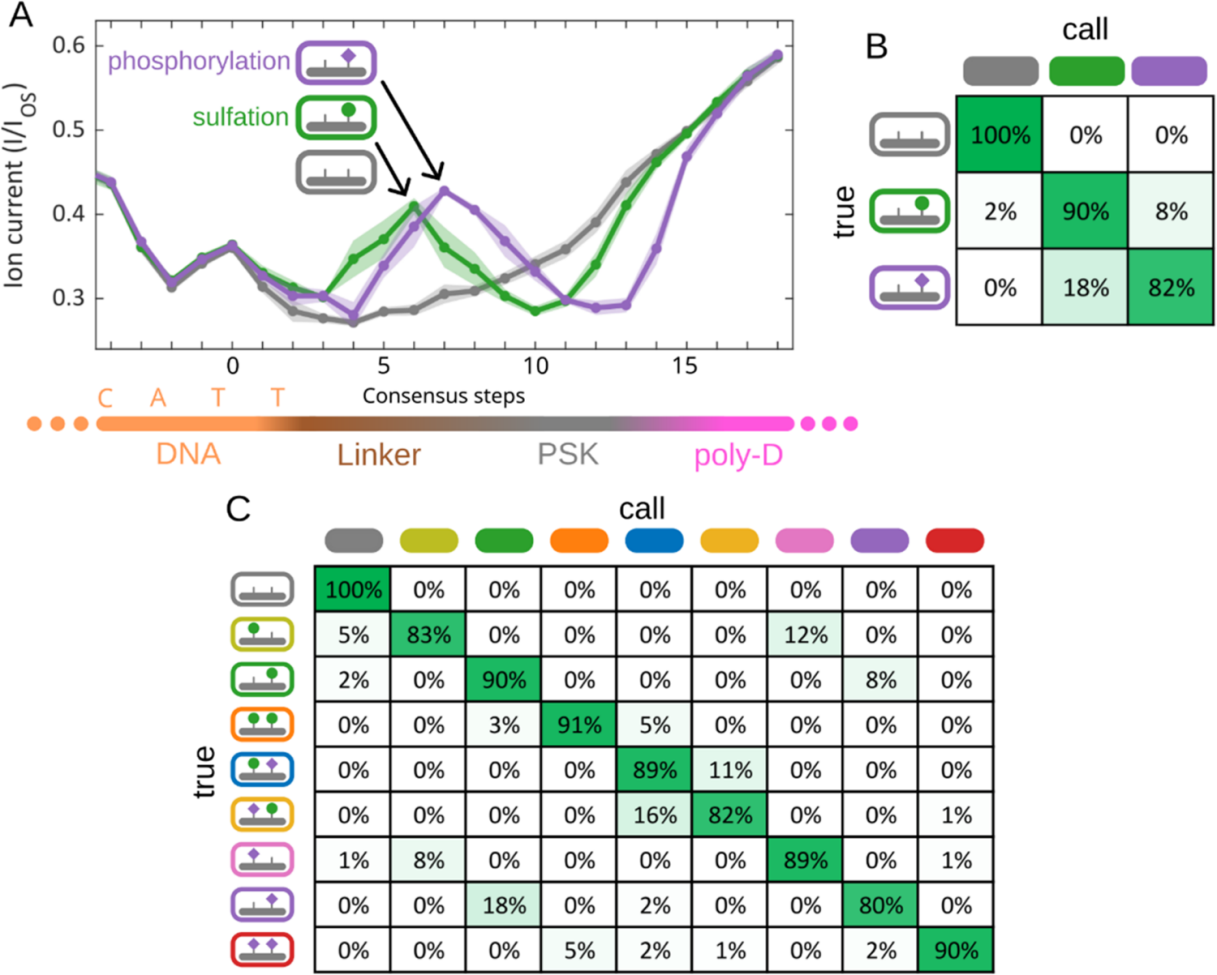
Sulfation and phosphorylation PTMs on the PSK hormone can be accurately identified. (A) Signal consensus traces from the unmodified PSK pentapeptide (gray), single sulfation (green), and single phosphorylation (purple). The starting DNA signals (orange region at the left) are identical across variants. Signal variation starts when the linker approaches the constriction site. The peptide signal is heavily influenced by the negatively charged poly-D tail (pink) that displays the signature ionic current ramp at the end of the traces. Means and standard deviations of the consensus steps are plotted as dots and shaded areas. (B) Confusion matrix for three variants of panel (A). Rows describe the true labels and columns describe the call results. (C) Confusion matrix for all PTM variants of the pentapeptide. This describes how molecules from the same “row” distribute across the “columns”. From top to bottom rows, the numbers of single-molecule events are 96, 130, 97, 115, 89, 134, 150, 132, and 175.

We found that we can robustly distinguish signal traces from different PTM variants of the PSK hormone. Upon collecting many single-molecule events (∼100 traces for each sample), a consensus signal was constructed for each PTM variant through an improved Baum–Welch hidden Markov model solver based on previous works (see Methods Section and Supporting Methods ).^15,26^ The different PTM states of the YIYTQ pentapeptide yielded significantly different consensus traces (Figure 2A). Phosphorylation on the 2nd tyrosine induced a pronounced signal peak in the middle of the consensus, similar to previous observations in phosphorylated immunopeptides.^15^ Sulfation on the same tyrosine residue similarly produced a signal peak but with a lower amplitude and an earlier onset when compared with phosphorylation (Figure 2A). This result is consistent with local salt modulation and charge-induced stretching of the polymer.^15,30^ The dianionic phosphotyrosine versus monoanionic sulfotyrosine induces a higher local salt concentration and generates stronger stretching and accordingly a delayed and higher signal peak. Notably, it is not surprising that the modified amino acid affected multiple consecutive ionic current values and thus resulted in a broad bump. Because of the Brownian motion and a finite-sized MspA constriction site, the measured ionic current at one point is affected by several residues. The term “*k*-mer”, originated from DNA sequencing methods,^31^ has been used to describe the number of residues that contribute to the signal convolution. For nanopore DNA sequencing, *k*-mer values of 4–6 nucleotides have been reported,^28^ corresponding to 8–12 Hel308 steps. Without a uniformly charged backbone, the peptide translocation will occur in a nonlinear manner,^15^ further convoluting the signal.

These distinct patterns from the sulfation and phosphorylation consensus resulted in highly accurate (>90%) variant identification of individual reads (Figure 2B, see Methods Section). Only a handful of the phosphorylated molecules were mistaken for sulfated molecules, mostly due to finite measurement noise. In contrast to the effects of the PTM on the 2nd tyrosine, peptide variants with only the modified 1st tyrosine showed less pronounced differences (Figure S5), which nevertheless could be mutually well-distinguished (91%) in variant calling.

Expanding variant identification across all possible PTM permutations highlighted the robustness of our nanopore detection method. Samples from the double, single, or unmodified variants could be well distinguished, with a consistently high accuracy varying between 80 and 100% (Figure 2C). The relatively more challenging variant callings occurred between single sulfation or phosphorylation on the same site as well as for double-modification samples. This prompted us to further dissect the PTM locations and charges on the pentapeptide.

### Location of the PTMs Can be Accurately Identified

**Figure 3:**
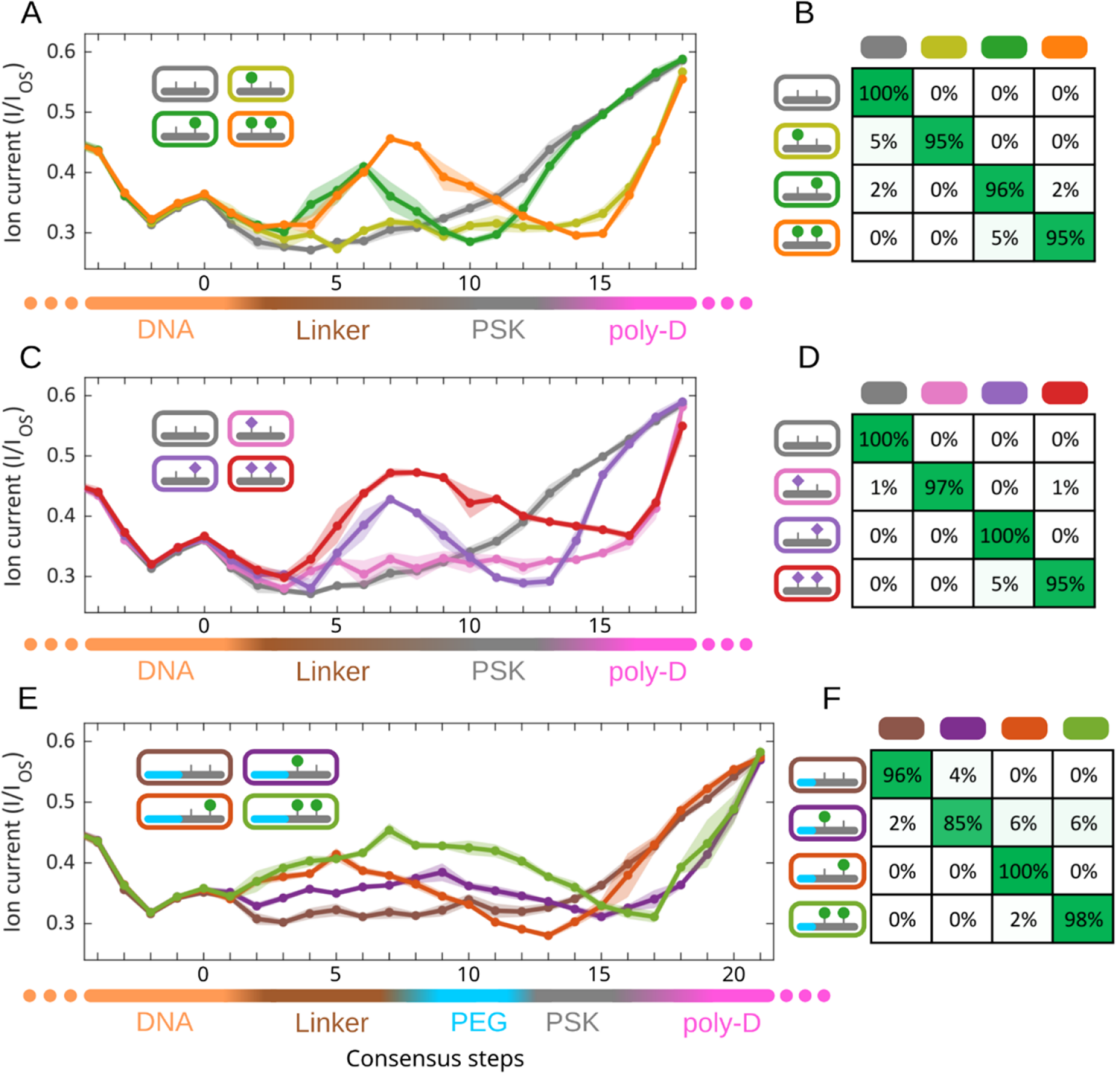
PTMs on two close-by tyrosine residues are well distinguished. (A) Consensus traces for the sulfation variants. The early signal peaks are related to the 2nd tyrosine PTM, while the tail pattern is related to the 1st tyrosine PTM. (B) Confusion matrix of the sulfation variants. (C) Consensus traces for the phosphorylation variants. (D) Confusion matrix of the phosphorylation variants. (E) Consensus traces for the sulfation variants with PEG insertion. The PEG linker extended the consensus by a few steps, and the signal differences were slightly attenuated. (F) Confusion matrix of the PEG-inserted sulfation variants. From top to bottom, *n* = 103, 124, 122, 106.

We found that it is possible to accurately identify the location of the PTM within the peptide, i.e., whether the same PTM occurred on the first or on the second tyrosine residue on the PSK. As Figure 3A shows, the four permutations of the pentapeptide carrying sulfation at two tyrosine residues generated distinct consensus patterns, yielding excellent identification accuracies of 95 to 100% (Figure 3B). Two seemingly independent observations can be made from the respective tyrosine measurements. For the modified 1st tyrosine, the traces did not show an early signal peak but instead presented a delayed and steeper signal ramp at the end. For the modified 2nd tyrosine, an early signal peak appeared, but the tail ramp occurred later than for the unmodified pentapeptide, gradually converging with the unmodified peptide. Double modification on the peptide combined these effects of the two sites, with a slightly higher peak amplitude from the additional negative charge. The phosphorylation variants of the peptide consistently followed the same rules, with stronger amplitudes (Figure 3C–D), consistent with the higher charges from the phosphorylation PTM. Because of the distinct effects of the two tyrosine PTMs, the modified variants carrying two negative charges, from either one phosphate or two sulfate groups, can also be accurately identified (Figure S6).

While we thus establish excellent discrimination between sulfation and phosphorylation PTMs on PSK, the molecular determinants that underlie the peptide translocations are not evident *a priori*. For example, why does the signal peak from the modified 2nd tyrosine appear so early? This made us hypothesize special interactions between the molecular linker and the pentapeptide. We thus created a longer linker length between the DNA and peptide by inserting a PEG8 segment (see Figure S2 and Methods Section), aiming to disrupt the suspected interaction. Interestingly, the permutations of sulfation in the PEG8 variants conformed to the same patterns as observed before, i.e., an early peak from the modified 2nd tyrosine and a delayed tail from the modified 1st tyrosine, albeit with slightly attenuated current differences (Figure 3E). This means that the “interaction” does not depend on the linker. As PEG8 is just upstream of the peptide in terms of translocation, the insertion also revealed a shifted current pattern corresponding to the peptide translocation (Figure S7, cyan shade). This indicates that, surprisingly, the signal peak induced by PTM on the 2nd tyrosine happens earlier than the translocation of the modified residue, suggesting some specific pore interactions on the 2nd tyrosine that depend on its PTM state.

### Charged Residues Dominate Nanopore Signals during Peptide Translocation

**Figure 4:**
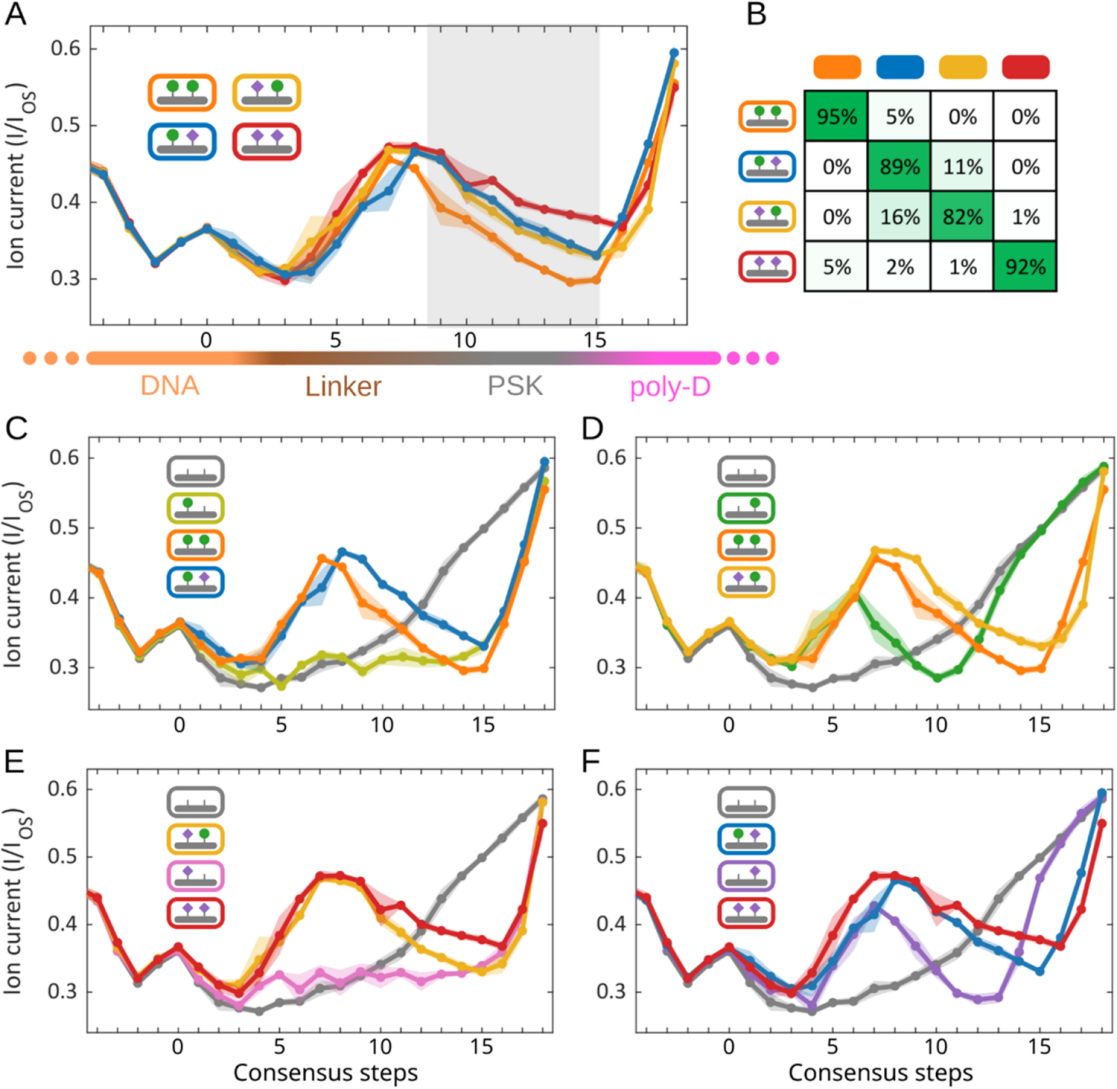
Charged residues on the peptide dominate nanopore signals. (A) Consensus traces for the double-modification variants. The shaded area highlights the steps with the largest current difference where the pentapeptide translocates the nanopore constriction. (B) Confusion matrix of the double-modification variants. The most difficult calling is between the sY-pY and pY-sY isomers. (C–F) Signal consensus of variants carrying sulfation on 1st tyrosine (A), sulfation on 2nd tyrosine (B), phosphorylation on 1st tyrosine (C), and phosphorylation on 2nd tyrosine (D). The unmodified peptide (gray) is used as a reference trace in all plots. Higher negative charge on the peptide leads to a higher current in general.

Subsequently, we examined the effects of the PTM states on each tyrosine residue. Because the ionic current through MspA is very sensitive to charges near the constriction site, we infer that the region with the largest current difference in the traces correspond to steps when modified tyrosine residues translocate through the pore constriction. The double-modification variants are expected to exhibit similar interactions, and they indeed all showed an early signal peak with comparable amplitudes during linker translocation (Figure 4A). The consensus steps with the largest current difference highlighted the same region (Figure 4A, gray area, steps 9–15) as the PEG8 samples for peptide translocation. Here, as the negative charge on the peptide increases, either by adding another PTM or by switching from sulfation to phosphorylation, a higher ionic current is obtained (Figure 4A, grey area). The current differences were sufficient to enable highly accurate variant calling (Figure 4B). The two variants with one sulfation and one phosphorylation resulted in almost identical consensus traces, but their shifted peak positions based on the phosphorylated residue demonstrated our high detection sensitivity, even for the smallest differences. Comparing variants with one fixed PTM further highlighted the two seemingly independent effects during early or late peptide translocation (Figure 4 C–F).

The tail pattern depends on whether the 1st tyrosine carries any PTM or not. This could be explained by hydrophobic interactions between the unmodified 1st tyrosine and the pore inner surface, similar to the observations described in a previous study,^26^ leading to higher current during translocation (Figure S8, No PTM). A charged PTM on this residue, such as sulfation or phosphorylation, disrupts this hydrophobic interaction and leads to a lower current due to the smaller conductive volume during translocation. For the earlier peak observed with a PTM on the 2nd tyrosine, molecular dynamics simulation indicated that this PTM has a higher propensity to engage in a transient charge interaction with the positively charged arginine residues at the bottom of the MspA (Figure S9). This suggests that the early signal peak is not related to the linker but is more likely due to the closer proximity of the 2nd tyrosine to the negatively charged poly-D tail (Figure S8, Y3 PTM). Recent nanopore studies also described ionic current alterations from charge interactions near the nanopore constriction site.^22,32^ Because of the transient nature of this interaction, the exact mechanism underlying the early signal peak remains difficult to dissect, highlighting the current challenges for interpreting nanopore signals from peptide measurements.

## Conclusions

In this study, we applied single-molecule nanopore sequencing to detect and distinguish post-translational modifications with an isobaric mass on the plant peptide hormone phytosulfokine (PSK). While functional plant peptide hormones often carry sulfated tyrosine residues and act at extremely low concentrations in plants,^6^ instability of tyrosine sulfation in typical mass spectrometry workflows has hindered investigation on this important family of peptides, especially when it comes to determining the site of modification.^9^ We demonstrated that single-molecule nanopore measurements can be done with mild sample preparation conditions and enable very accurate determination of sulfated and phosphorylated sites on peptides. Single PTM, either sulfation or phosphorylation on the peptide, generated clearly distinguishable signal patterns. Permutations of PTMs on the two tyrosine residues revealed a surprising pattern where modifications on the 2nd tyrosine gave rise to more pronounced signal changes, emphasizing the dominant influence of charged residues on ionic current. The two distinct effects of the PTMs on the respective tyrosine residues allowed for very accurate variant identification of these proximal modifications. Even with single reads, our work demonstrates that nanopore measurements can provide an extremely high distinguishing power of less than 1 Da. The single-read accuracies range from 80 to 100%, which can be further improved to basically 100% by rereading the same individual peptide multiple times by adjusting the experimental conditions.^15^

More generally, the current findings show the extraordinary strength of nanopore methodologies for PTM detection, in particular, for functionally important short peptides. Once measurement references are established, variant detection is readily attainable at the highest distinguishing power, as is clear from our data, where we demonstrated the excellent distinguishing power between even two closely positioned and very similar PTMs as sulfation and phosphorylation. The experimental workflow is carried out under physiological conditions, without harsh sample treatment, preserving chemically labile PTMs, such as sulfation. These mark major advantages over mass spectrometry for position-specific PTM detection. With a generic peptide conjugation strategy for DNA attachment available^33^ and a careful sample preparation workflow,^34,35^ our nanopore methodology can be widely applied to many native peptide samples, such as peptide hormones and neuropeptides.

## Methods

### SPAAC Peptide–Oligonucleotide Conjugation

BCN-modified DNA was custom synthesized and purchased from Thermo Fisher Life Sciences with UHPLC-MS quality control performed in-house (Figure S10). The desired peptide at 1.3 mM (20 nmol) and BCN-DNA at 0.3 mM (5 nmol) were added to a 0.5 mL microcentrifuge tube and reacted in Mili-Q water overnight at room temperature. Samples were purified with Amicon ultraspin filtration units with 10 kDa molecular weight cutoff (MWCO) using phosphate buffer (50 mM sodium phosphate, pH 6.0). Obtained samples were analyzed with RP-UHPLC-MS (Figures S11–S23). Concentrations were measured with a Nanodrop spectrophotometer and corrected for the extinction coefficient of the template DNA as determined by the supplier. Samples were aliquoted, snap-frozen, and subsequently lyophilized.

### Solid-Phase Peptide Synthesis of PSK-Like Peptides

Amino acids and peptide synthesis reagents were purchased from Novabiochem. Modified amino acids, Fmoc-Tyr(SO_2_ONp)–OH and Fmoc-Tyr(PO(OBzl)OH)–OH, were purchased from Merck Life Science. Azidoacetic acid was purchased from TCI Europe. Azido-PEG_8_-NHS ester was purchased from Broadpharm. Peptides were synthesized following standard Fmoc/*t*Bu solid-phase peptide synthesis (SPPS) strategy in a split method^25^ (see Supporting Information). Peptides were cleaved from the resin with different trifluoroacetic acid (TFA) cocktails, depending on the N-terminal modification. Treatment of peptide with 2 M NH_4_OAc at 45 °C for 40 h resulted in neopentyl removal from the sulfated tyrosine residues. Obtained deprotected peptides were purified by preparative HPLC and analyzed with UHPLC-MS for quality control (Figures S11–S23).

### Nanopore Measurements

Nanopore experiments on DNA-peptide conjugate molecules were performed as described in previous studies.^15,26^ DPhPC lipids were purchased from Avanti Polar Lipids (SKU: 850356C). M2-MspA mutant was purified by Genscript. Hel308 used in this study is from *Thermococcus gammatolerans* (accession number WP_015858487.1) and was purified in-house. Teflon apertures on custom U-tube devices were painted with DPhPC lipids to form bilayers submerged in buffer H (400 mM KCl, 10 mM HEPES, pH 8.00). Cross-membrane voltage was set to 180 mV under the control of a National Instruments X series DAQ and operated with custom LabVIEW software. Around 0.5 μL (1 μg/mL) of MspA was added to the *cis* well until a signature 140–150 pA ionic current increase was observed, indicating single nanopore insertion. The *cis* well is then perfused with buffer H supplemented with 1 mM ATP and 10 mM MgCl_2_. Hel308 and conjugate molecules were added to the *cis* well to the final concentrations of around 100 and 10 nM, respectively. At 37 °C, ionic current data were acquired at 50 kHz sampling frequency using an Axopatch 200B patch clamp amplifier and filtered with a 10 kHz 4-pole Bessel filter. Data on each sample in this study (see Table S1) are collected separately from individual nanopore experiments, with different MspA nanopore molecules from the same expression batch.

### Data Processing of Single-Molecule Events

Ionic current recordings were first downsampled to 5 kHz, and translocation events were identified by thresholding the current with the open pore current. The open-state current (*I*_OS_) of the nanopore is used to normalize the current blockades across different translocation events. The Hel308-mediated translocation events of conjugate molecules, containing both DNA and peptide signals, were selected by eye based on second-long duration and known DNA signal patterns. The single-molecule translocation event is computationally cut at the end of the predicted DNA signals (Figure 1D, derived from a 6-mer DNA model^28^) to consistently separate the DNA and non-DNA segments. Signal steps generated by the helicase movement were extracted by a change point algorithm, described previously.^28^ These ionic current steps were filtered by excluding any levels outside the bounds of expected current values (*I*/*I*_OS_ < 0.15 or *I*/*I*_OS_ > 0.7) before being linearly calibrated by aligning to a fixed DNA prediction reference (similar to Figure 1D). The calibrated signal steps from the non-DNA segment (linker and peptide) were then trimmed at the end by thresholding the tail ramp at 0.6 relative current.

### Consensus Generation and Variant Calling

The events from each peptide variant were randomly split into two equal groups: one for consensus generation and one for variant calling. Signal steps in the linker-peptide region were manually picked in a selection of typical events (*n* = 4–6) to approximate the stepwise current levels generated by the helicase without back-stepping or skipping. This process produced some good input guesses for the hidden Markov model solver for the peptide consensus. Along with the peptide events in the consensus generation group, the input guess was used in a custom Baum–Welch script to solve the hidden Markov model using a maximum-a posteriori likelihood (MAP) algorithm (see Supporting Information). This assigns likelihood values that each of the signal steps in the event was produced by a particular true template position within the constriction (helicase step number).^26^ The resulting model is the consensus for the peptide variant with mean and standard deviation values for each helicase step. A forward–backward alignment algorithm was developed to support weighting in the Baum–Welch process and provide variant calling assessment (see Supporting Information). For the linker-peptide events in the variant calling group, they carry a “true” label based on the measurement of the pure sample. Each event was aligned to all of the consensus within the variant calling group for a series of alignment likelihoods. The consensus alignment with the highest likelihood was designated as the “call” label. The accuracy of the variant call was the percentage of call labels matching the true labels of the peptide events within the variant calling group.

### COMSOL Simulation

Numerical simulations of the MspA-helicase-peptide system were implemented in COMSOL Multiphysics 5.4 with a two-dimensional axial symmetrical domain. The simulations included the fluid domain, the lipid membrane, MspA nanopore, helicase, and the DNA-linker-peptide strand. With the contour of the M2-MspA nanopore (PDB: 1UUN), the charged residues in the inner wall of the nanopore protein were marked at positions of 63 (−), 118 (+), and 134 (+) (Figure S24). The DNA-linker-peptide strand was approximated with cylindrical columns with corresponding thicknesses. The single-strand DNA (ssDNA) carried −1e/nucleotide, the linker was neutral in charge, and the D_15_ tail carried −1e/amino acid. The charge of tyrosine residues was set at 0, −1e, and −2e, for unmodified, sulfated, and phosphorylated states. To calculate the ionic current, ion flux was integrated on the cross-sectional area located at the narrowest restriction of the nanopore. The relative current is based on the highest current during translocation. See Supporting Information for detailed descriptions.

## Data Availability

The raw data, processed data, processing scripts, analysis scripts, simulation project files, and plotting scripts for reproducing the figures are deposited in Zenodo (doi: 10.5281/zenodo.11199977).

## Supporting Information Available

Additional details on chemical synthesis of DNA-peptide conjugates, COMSOL simulation, MD simulation, and consensus generation (Supporting Methods); sequence information on DNA and conjugates used in this study (Table S1); mass spectrometry and HPLC data on DNA-peptide conjugates (Figures S1 and S10–S23); details of the DNA-peptide conjugate chemical structure (Figure S2); example of raw traces of the nanopore recording (Figure S3); details on COMSOL simulation (Figures S4 and S24); more consensus comparisons (Figures S5–S7); proposed molecular interactions during translocation (Figure S8); MD simulation on possible charged interactions (Figure S9) (PDF)

## Notes

**Preprint Statement** This work has been previously submitted to a preprint server.^36^
